# Thalamic Reticular Nucleus Parvalbumin Neurons Regulate Sleep Spindles and Electrophysiological Aspects of Schizophrenia in Mice

**DOI:** 10.1038/s41598-019-40398-9

**Published:** 2019-03-05

**Authors:** Stephen Thankachan, Fumi Katsuki, James T. McKenna, Chun Yang, Charu Shukla, Karl Deisseroth, David S. Uygun, Robert E. Strecker, Ritchie E. Brown, James M. McNally, Radhika Basheer

**Affiliations:** 1 VA Boston Healthcare System and Harvard Medical School, Dept. of Psychiatry, West Roxbury, MA USA; 2 Stanford University, Psychiatry and Behavioral Sciences/Bioengineering, Stanford, CA USA

## Abstract

The thalamic reticular nucleus (TRN) is implicated in schizophrenia pathology. However, it remains unclear whether alterations of TRN activity can account for abnormal electroencephalographic activity observed in patients, namely reduced spindles (10–15 Hz) during sleep and increased delta (0.5–4 Hz) and gamma-band activity (30–80 Hz) during wakefulness. Here, we utilized optogenetic and reverse-microdialysis approaches to modulate activity of the major subpopulation of TRN GABAergic neurons, which express the calcium-binding protein parvalbumin (PV), and are implicated in schizophrenia dysfunction. An automated algorithm with enhanced efficiency and reproducibility compared to manual detection was used for sleep spindle assessment. A novel, low power, waxing-and-waning optogenetic stimulation paradigm preferentially induced spindles that were indistinguishable from spontaneously occurring sleep spindles without altering the behavioral state, when compared to a single pulse laser stimulation used by us and others. Direct optogenetic inhibition of TRN-PV neurons was ineffective in blocking spindles but increased both wakefulness and cortical delta/gamma activity, as well as impaired the 40 Hz auditory steady-state response. For the first time we demonstrate that spindle density is markedly reduced by (i) optogenetic stimulation of a major GABA/PV inhibitory input to TRN arising from basal forebrain parvalbumin neurons (BF-PV) and; (ii) localized pharmacological inhibition of low-threshold calcium channels, implicated as a genetic risk factor for schizophrenia. Together with clinical findings, our results support impaired TRN-PV neuron activity as a potential cause of schizophrenia-linked abnormalities in cortical delta, gamma, and spindle activity. Modulation of the BF-PV input to TRN may improve these neural abnormalities.

## Introduction

The cortico-thalamic network is centrally implicated in a number of fundamental brain processes including sensory perception, pain, attention, consciousness, and sleep/wake^1^. The thalamus and cortex work synergistically through a highly complex array of reciprocal connections to serve these functions^2–4^. Proper regulation of these connections and their functional output requires an exquisite level of inhibitory control^5^. Abnormal cortico-thalamic network dynamics have been reported in a number of neurologic and psychiatric disorders, including schizophrenia^4,6,7^. However, the pathophysiology of these abnormalities is not well understood. Schizophrenic patients consistently exhibit reductions in the density of sleep spindles, which are brief rhythmic events (10–15 Hz) evident during non-rapid eye movement (NREM) sleep^8^. These spindle abnormalities are implicated in impaired sleep-dependent memory consolidation^9,10^, and may represent an endophenotype for schizophrenia which contributes to cognitive symptoms^8^. Lesion and deafferentation experiments suggested that the thalamic reticular nucleus (TRN) is the subcortical generator of sleep spindles^11–13^. Thus, reduced activity of TRN neurons is an attractive but untested hypothesis, to explain sleep spindle abnormalities in schizophrenia and other disorders.

Several recent studies support this idea. First, postmortem evidence from patients with schizophrenia has revealed reductions in the levels of two activity-dependent markers in TRN GABAergic neurons, the calcium binding protein parvalbumin (PV) and extracellular matrix structures called perineuronal nets^14^. Furthermore, mutations in the crystallin βB2 gene associated with schizophrenia reduce TRN-PV neuron density and are associated with schizophrenia-like impairment in pre-pulse inhibition^15^. Finally, genetic studies^16,17^ have implicated *CACNAI1*, a gene encoding the low-threshold calcium channel Ca_V_3.3 which is highly expressed in TRN and responsible for the rebound burst discharge in TRN neurons during sleep^18^, as a schizophrenia risk gene. Another recent study showed that deletion of the schizophrenia-associated gene, ErbB4, from TRN somatostatin (SOM) neurons impairs sensory selection in mice^19^. These findings suggest that TRN GABA/PV and GABA/SOM neurons, like their cortical counterparts^20^, may exhibit reduced activity in schizophrenic patients. However, whether changes in the activity of TRN neurons can account for cortical electroencephalogram (EEG) abnormalities in this disease is unclear.

Unlike the burst discharge typical of TRN neurons associated with sleep spindles^21^, during wakefulness, TRN neurons discharge at gamma-band frequencies (30–80 Hz) and gate sensory transmission to the cortex^22^. Thus, reduced activity of TRN neurons may also contribute to the widely reported impaired EEG gamma-band (30–80 Hz) response to auditory stimuli in schizophrenia^23^. Blockade of NMDA receptors in TRN leads to increased delta (0.5–4 Hz) activity^24^, resembling findings in patients^25^. Therefore, reduced TRN activity may contribute to altered activity in several frequency bands in schizophrenia. However, the role of specific subsets of TRN neurons in controlling cortical EEG during wakefulness and sleep remains to be further elucidated. Here, we focus on our optogenetic studies on the major subset of TRN neurons which contain PV.

Another important unexplored area is how extra-thalamic inhibitory inputs shape the output of the TRN and the larger cortico-thalamic network^5^. Afferent inputs to the TRN arise from a number of neuromodulatory systems in the brainstem and basal forebrain (BF). Our previous studies^26,27^ focused on GABAergic BF-PV neurons, which project directly to the cortex as well as to TRN^28,29^. Thus, here we investigate the role of this source of inhibitory input to TRN on both spindle generation and sleep/wake behavior, as a potential novel therapeutic target for addressing cortico-thalamic dysrhythmias.

In this study we developed and validated a novel, low power, waxing-and-waning optogenetic stimulation paradigm which, unlike a single pulse laser stimulation used by us (this study) and others^30^, induced spindles that were indistinguishable from spontaneously occurring sleep spindles that are detected by an automated algorithm which was developed and validated in-house^31^. We show that direct optogenetic inhibition of TRN-PV neurons elevated background delta and gamma activity and thereby impaired the 40 Hz auditory steady-state response. Finally, for the first time we show that sleep spindle density is reduced by activation of a major inhibitory input to TRN arising from BF-PV neurons as well as by localized inhibition of the T-type calcium channels in TRN, implicated as risk factors by genetic studies, using intracerebral reverse microdialysis infusion of the selective T-type inhibitor TTA-P2.

## Results

### **W**axing-and-Waning Optogenetic Stimulation of TRN-PV Neurons Reliably Elicits Sleep Spindles

**Figure 1:**
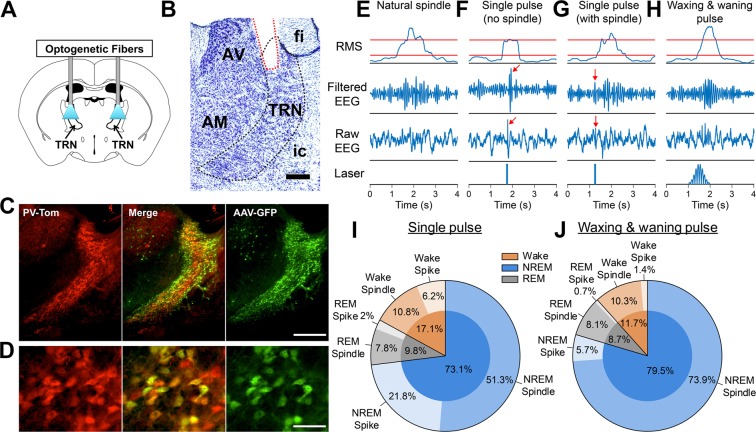
Waxing-and-waning optogenetic stimulation of thalamic reticular nucleus (TRN) parvalbumin (PV) neurons elicits cortical electroencephalographic (EEG) events indistinguishable from spontaneously occurring sleep spindles. (**A**) TRN-PV neurons were bilaterally transduced with AAV-ChR2-EYFP and optogenetically stimulated. Coronal schematic representation of TRN image credit: Allen Institute, adapted from The Allen Mouse Brain Atlas (Available from: http://mouse.brain-map.org)^71^. (**B**) Representative cresyl violet staining confirmation of optogenetic fiber placement (red) in TRN. Staining did not show cellular damage due to laser application, which was consistent across all cases evaluated. Abbreviations: AV, anteroventral thalamic nucleus; AM, anteromedial thalamic nucleus; fi, fimbria of hippocampus; ic, internal capsule; TRN, thalamic reticular nucleus. Scale bar: 250 µm. (**C**,**D**) Confirmation of viral transduction efficiency and selectivity. AAV-GFP (green) was bilaterally injected into TRN of 2 PV-tdTomato (red) mice. Three coronal sections per animal representing rostral, medial, and caudal TRN were bilaterally analyzed for AAV-GFP/PV-tdTomato co-localization. 83 ± 3% of AAV-GFP transduced neurons co-expressed td-Tomato, suggesting high selectivity. 62 ± 2% of TRN-PV-tdTomato neurons were transduced by AAV-GFP, indicating high efficiency. Scale bars: 500 µm (top), 25 µm (bottom). (**E**–**H**) Spindles were detected as brief elevations in 10–15 Hz oscillatory activity in cortical EEG using a custom-built MATLAB script (see Methods). Events were counted as spindles if the root mean square (RMS) transform of band pass filtered EEG data exceeded an upper threshold (top red line, 3.5x mean RMS power) and the duration was >0.5 s as determined by crossing of a lower threshold set at 1.2x mean RMS power (bottom red line). Panel E shows an example of a spontaneously occurring (natural) spindle, while the panels F, G, & H show detected events elicited by optogenetic stimulation of TRN-PV neurons during non-rapid-eye-movement (NREM) sleep. We utilized a single pulse paradigm (**F**,**G**) and a more complex waxing-and-waning stimulation protocol (**H**). While both paradigms resulted in an elevation of detected spindle-like events, the events generated by the waxing-and-waning protocol were more morphologically similar to physiological spindles. The single pulse paradigm at times generated sharp EEG spike-like events (denoted by red arrows). (**I**,**J**) To characterize the single pulse laser paradigm vs. the waxing-and-waning paradigm, laser was repeatedly applied every 10 s regardless of animal’s state (without NREM gate) during the 3-h recordings. The proportion of optogenetically induced spindles in each state, including both spindle-like and spike-like spindles, was calculated by dividing the number of induced spindles in a specific state by a total number of induced spindles from all states (the inner circles of the charts). A higher percentage of induced spindles in wakefulness was observed with the single pulse laser paradigm compared to the waxing-and-waning paradigm (**I**,**J**, orange). The waxing-and-waning paradigm showed higher efficiency in eliciting spindles during NREM sleep compared to wakefulness (**J**, blue and orange). The proportion of spindle-like spindles and spike-like spindles was also calculated for each state and shown in the outer circles of the charts. The waxing-and-waning paradigm reduced the sharp spike-like EEG events compared to the single pulse paradigm.

To test the sufficiency of TRN-PV neurons for the generation of sleep spindles, and to identify an effective stimulation paradigm, we expressed channelrhodopsin (ChR2) in TRN-PV neurons (n = 5, Fig. 1A,B). Control experiments confirmed high selectivity and efficiency of viral transduction in TRN-PV neurons (Fig. 1C,D). Mice were stimulated bilaterally once every 10 s for 4 hours (zeitgeber time, ZT2–8), targeting the rostrodorsal region of the TRN, which sends substantial projections to thalamic relay neurons projecting to frontal cortices^32,33^, where our EEG recording electrodes were located. To minimize changes in state, the laser power was titrated, for each animal, to the lowest level (0.5–1.5 mW) eliciting a change in EEG but not electromyography (EMG). We initially tested whether a single pulse (10 ms) of TRN-PV stimulation, similar to that employed in prior studies^30,34^ could induce cortical EEG events similar to spontaneously occurring sleep spindles (Fig. 1E). This protocol (n = 3) increased the probability of detected spindle-like EEG events within 2 s of stimulation (32.5 ± 5.1% of trials vs. 16.4 ± 1.1% with sham stimulation). However, in ~30% of trials, this single-pulse protocol also produced sharp, EEG spikes immediately after laser onset (average latency to spike peak 26 ± 3 ms), with a temporal profile unlike spindles. These EEG spikes were observed either alone or followed by a spindle-like event (Fig. 1F,G, respectively).

To avoid these spike-like events, we developed a 10 Hz waxing-and-waning optical stimulation protocol (1 s), modeled after the natural time course and profile of spindles (Fig. 1H). This stimulation pattern was generated using a Gaussian envelope transform of a train of 100 ms half-sinewaves, for analog modulation of laser power. This paradigm led to a significant increase in the percentage of optogenetically-induced EEG events with a profile essentially indistinguishable from spontaneously occurring spindles (Fig. 1E,H). Directly comparing this novel protocol to the single-pulse paradigm described above, without restricting the laser application to the specific sleep-wake states, we also observed a marked reduction in spike-like events (Sum of all spike-like events with single pulse: 30% vs. waxing-and-waning pulse: 7.8%, n = 3; Fig. 1I,J), especially evident during NREM sleep (Single pulse 21.8% vs. Waxing-and-waning pulse: 5.7%). The waxing-and-waning paradigm was also highly efficient in eliciting spindles during NREM sleep when compared to the single-pulse paradigm (Single pulse 73.1% vs. Waxing-and-waning pulse: 79.5%). To further increase the NREM-specificity for inducing spindles, we utilized the NREM detection system (NREM gate) with the waxing-and-waning paradigm for further TRN ChR2 experiments.

**Figure 2:**
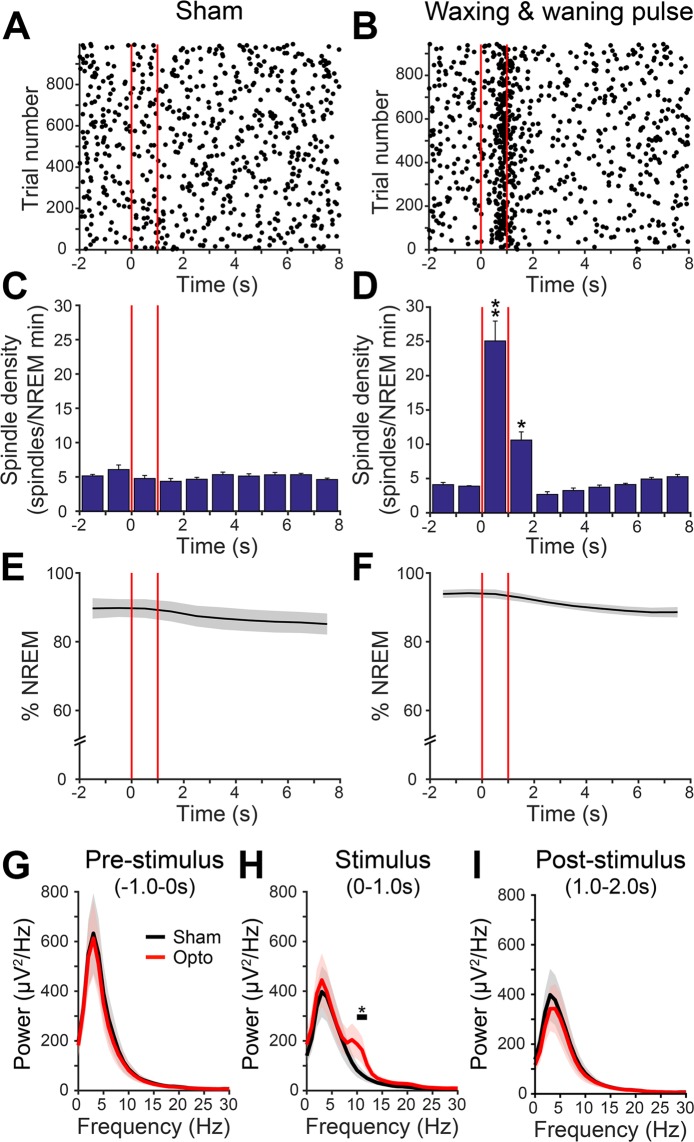
NREM-gated optogenetic waxing-and-waning stimulation of thalamic reticular nucleus (TRN) parvalbumin (PV) neurons increases cortical spindle density. (**A**,**B**) Representative raster plots of detected spindle-like events produced by optogenetic or sham stimulation (red lines) relative to non-rapid-eye-movement (NREM) sleep show a pronounced elevation in spindle density with optogenetic stimulation. Optogenetic waxing-and-waning stimulation was applied for 1 s with a minimum 9 s interval between stimulation trials repeatedly over 6 h of recording time. Stimulation was restricted to NREM sleep using a NREM detection system. (**C**,**D**) Histogram depiction shows a significant increase in averaged NREM spindle density with optogenetic stimulation when compared to sham (n = 5, ANOVA, **p < 0.01; *p < 0.05). There was no immediate arousal observed with optogenetic or sham stimulation (**E**,**F**). EEG power spectral density analysis of the 1 s pre-stimulus period (**G**) showed high delta activity typical of the NREM sleep state prior to both sham (black) and optogenetic stimulation (red) due to the use of the NREM detection system capturing high delta activity. (**H**) During the 1 s stimulus period, there was a statistically significant increase across the 10–12 Hz peak spindle frequency band with optogenetic stimulation (n = 5, Jackknifing U-Statistic, *p < 0.05) compared to sham. (**I**) No significant difference in EEG power was observed between sham and optogenetic stimulation during the 1 s post-stimulus period.

Overall (n = 5), NREM-specific stimulation of TRN-PV neurons with the waxing-and-waning protocol (1 s stimulation with a minimum 9 s interval between stimulation trials over 6 h of recording time) was extremely efficient, with spindles elicited on 58.6 ± 6.9% of laser trials (Fig. 1H). Additionally, we observed a significant increase in NREM spindle density associated with stimulation (Fig. 2A–D, spindles/min NREM, 25.1 ± 3.2, p = 0.003), while total NREM sleep was unaltered (Fig. 2E,F), and during the entire 6 h recording period (sham: 63.2 ± 2.5% vs. stimulation 61.4 ± 2.8%; p = 0.63). Spectral analysis of the EEG during the 1 s pre-stimulation period showed high delta (0.5–4 Hz) power typical of NREM sleep in both the sham and optogenetic stimulation conditions (Fig. 2G) due to our NREM gating system, which operated by monitoring elevations in EEG delta power in real time. Notably, a statistically significant increase in the peak 10–12 Hz spindle frequency range was observed only with optogenetic stimulation (n = 5, p < 0.05, Fig. 2H), but not in the pre- or post-stimulus periods (Fig. 2G,I).

### Intermittent Optogenetic Inhibition of TRN-PV Neurons Does Not Reduce Spindle Density, but Increases Wakefulness and Background Delta and Gamma Activity

**Figure 3:**
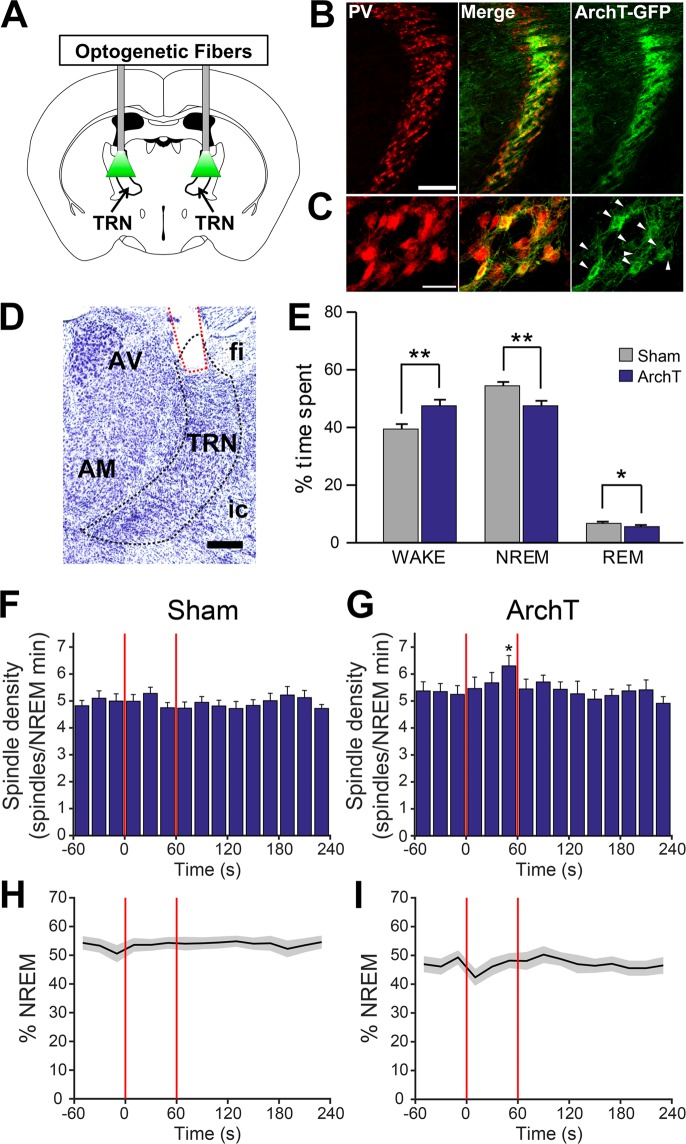
Optogenetic inhibition of thalamic reticular nucleus (TRN) parvalbumin (PV) neurons did not decrease spindle density but increased wakefulness and decreased non-rapid-eye-movement (NREM) sleep. (**A**) TRN-PV neurons were bilaterally transduced with AAV-ArchT-GFP and optogenetically stimulated 1 min every 5 min for 4 h total. Coronal schematic representation of TRN image credit: Allen Institute, adapted from The Allen Mouse Brain Atlas (Available from: http://mouse.brain-map.org)^71^. (**B**,**C**) Representative immunohistochemical confirmation of targeting and specificity of ArchT-GFP expression (green, right panels) in TRN-PV (red, left panels) neurons. Arrowheads indicate ArchT-expressing neurons colocalized with PV (also shown as green/red overlap in the middle panels). Scale bars: 500 µm (top), 25 µm (bottom). (**D**) Representative cresyl violet staining conformation of an optogenetic fiber placement (red) in TRN. Cellular damage due to laser application was not evident. Abbreviations: AV, anteroventral thalamic nucleus; AM, anteromedial thalamic nucleus; fi, fimbria of hippocampus; ic, internal capsule; TRN, thalamic reticular nucleus. Scale bar: 250 µm. (**E**) Overall, during the 4 h period of the experiment, there was a cumulative decrease in NREM/REM sleep and increase in wakefulness with ArchT stimulation. (**F**,**G**) Histograms for the averaged spindle density in 20 s bins show that, contrary to our predictions, ArchT-mediated inhibition progressively increased spindle density during 1 min of laser application when compared to sham (n = 15). (**H**,**I**) Compared to sham inhibition (**H**), during the period of ArchT stimulation there was a decrease in the percentage time spent in NREM sleep (**I**). *p < 0.05; **p < 0.001.

We next examined whether we could reduce sleep spindle density by optogenetic inhibition of TRN-PV neurons using the inhibitory proton pump, Archaerhodopsin (ArchT). *In vitro* recordings confirmed optogenetic hyperpolarization of ArchT-transduced TRN-PV neurons, which inhibited action potential discharge (Supplemental Fig. 1). *In vivo*, mice expressing ArchT bilaterally in TRN-PV neurons (Fig. 3A–D, n = 15) were optically inhibited for 1 min/5 min for 4 h from ZT3-ZT7, and effects were compared with sham inhibition, within animals. Unlike optogenetic stimulation, ArchT inhibition had a significant effect on behavioral state resulting in an overall increase in wakefulness (Sham: 39.3 ± 1.8%; ArchT: 47.5 ± 1.9%; p < 0.001; Fig. 3E). Surprisingly, ArchT inhibition of TRN-PV neurons did not reduce NREM spindle density (Fig. 3F,G). In fact, spindle density tended to increase toward the latter portion of the stimulus (final 20 s of 1 min laser period, sham: 4.72 vs. ArchT inhibition: 6.3 spindles/min, p < 0.01). Additionally, the onset of the ArchT stimulus was often associated with brief arousals, an effect not observed with sham inhibition (Fig. 3H,I), which likely contributed to the observed overall increase in wakefulness.

**Figure 4:**
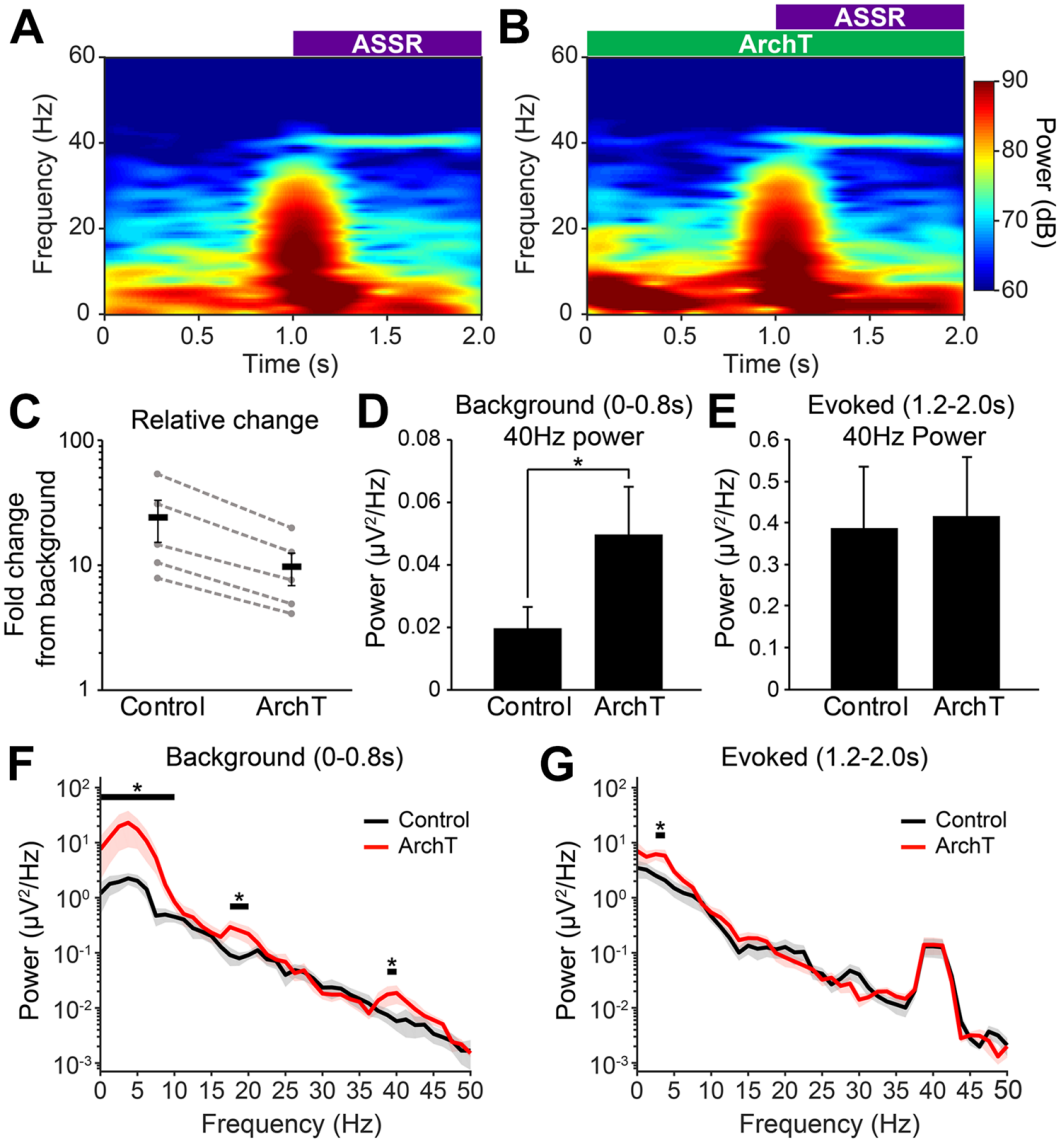
Optogenetic inhibition of thalamic reticular nucleus (TRN) parvalbumin (PV) neurons alters spontaneous electroencephalogram (EEG) activity leading to an abnormal auditory steady state response (ASSR). (**A**,**B**) Grand-average spectrograms of the 40 Hz ASSR under control conditions (**A**) and with bilateral ArchT inhibition of TRN-PV neurons (**B**). (**C**) Overall, optical inhibition of TRN-PV neurons using archaerhodopsin (ArchT) resulted in a trend level (p = 0.07) decrease in the relative change in 40 Hz ASSR power. (**D**) Background 40 Hz power prior to auditory stimuli (0–0.8 s) was significantly increased whereas (**E**) evoked 40 Hz power during the auditory stimuli (1.2–2 s) was unchanged. Thus, the reduction in the ASSR with ArchT inhibition of TRN-PV neurons is due to a significant (p < 0.05) increase in spontaneous activity prior to the auditory stimuli. (**F**) Further examination of the power spectral density during the ASSR task show that background activity is significantly elevated in power across the delta & theta (0.5–9 Hz), low beta (~18 Hz), and gamma (~40 Hz) bands with optical inhibition of TRN-PV neurons. (**G**) Evoked activity during the 40 Hz train of auditory stimuli shows only a small elevation in power in the delta range when compared to control. (n = 5, Jackknifing U-Statistics, *p < 0.05).

We also tested the effect of ArchT inhibition of TRN-PV neurons on spontaneous EEG activity and sensory-evoked gamma oscillations using the auditory steady-state response (ASSR) task. Here, we employed a different paradigm for ArchT inhibition from that described above. Mice (n = 6) were exposed first to a train (1 s) of 40 Hz auditory stimuli (85 db) alone, and then with combined ArchT inhibition (1 s before, and during auditory stimuli). This paradigm was repeated, providing a total of 120 trials for each experimental condition. The EEG response was averaged, and power spectral density analysis used to examine the effects on the power of cortical activity at 40 Hz (Fig. 4A–E). ArchT inhibition led to a trend level (p = 0.07; paired t-test) decrease in the relative change in 40 Hz ASSR compared to control (Control: 24.2 ± 8.6-fold increase; ArchT: 9.7 ± 2.9-fold increase; Fig. 4C). This deficit was due to a significant pre-stimulus elevation in background 40 Hz activity (Control: 0.019 ± 0.007 µV^2^/Hz; ArchT: 0.050 ± 0.016 µV^2^/Hz; p = 0.03; Fig. 4D), while no effect was observed on evoked 40 Hz activity (Control: 0.39 ± 0.15 µV^2^/Hz; ArchT: 0.42 ± 0.14 µV^2^/Hz; p = 0.32; Fig. 4E). Looking at the whole power spectrum, we also observed statistically significant elevations in background power in the delta (0.5–4 Hz), theta (5–9 Hz) and low beta (~18 Hz) frequency bands preceding auditory stimulation (Fig. 4F; p < 0.05). A small but significant increase was also observed in delta during auditory stimuli (Fig. 4G).

### Stimulation of the BF-PV Input to TRN Inhibits Spindle Generation and Increases Wakefulness

**Figure 5:**
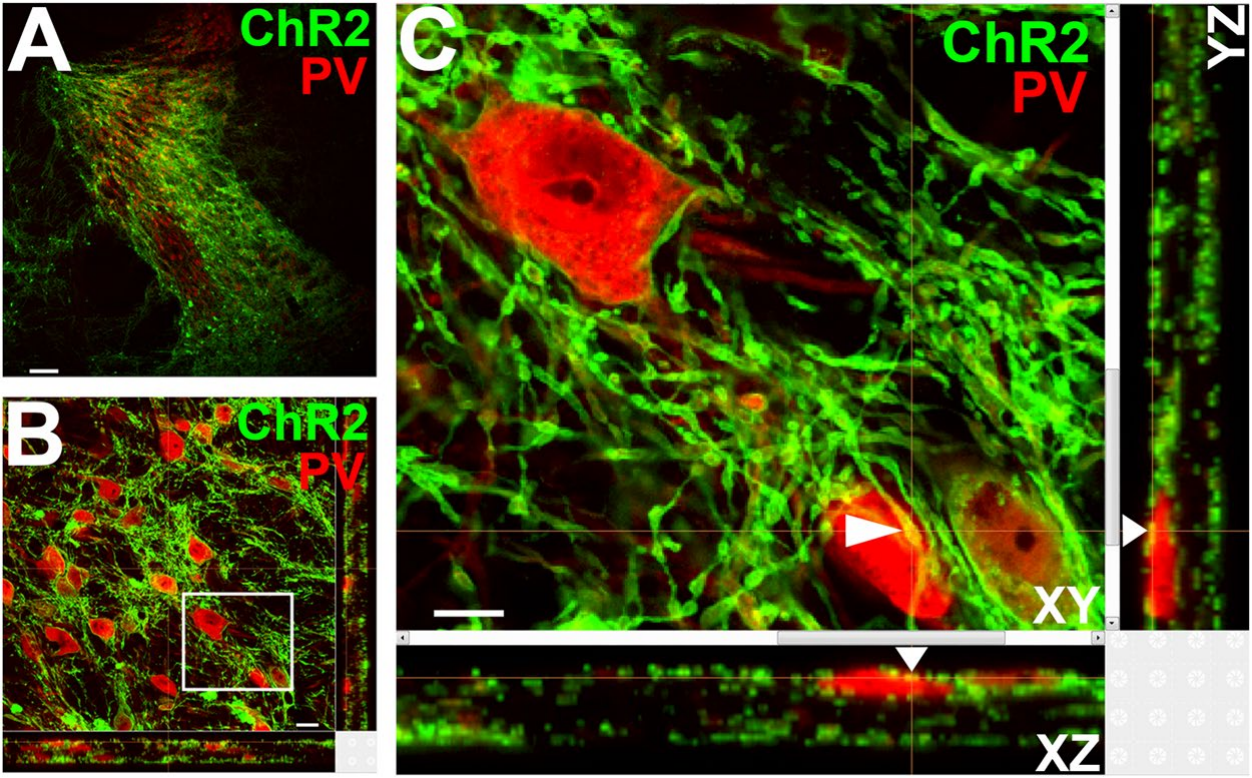
Transduction of basal forebrain (BF) parvalbumin (PV) neurons produced a dense plexus of channelrhodopsin2-enhanced yellow fluorescent protein (ChR2-EYFP) expressing fibers and terminals throughout the thalamic reticular nucleus (TRN) closely apposed to TRN-PV neurons. (**A**) Low-power (10x) image shows the dense innervation of the TRN (red PV neurons) by BF-PV axons (green). Scale Bar: 100 µm. (**B**) High-power (20x) confocal z-stack image (36 optical sections, 1 µm thickness) illustrates the close apposition of BF-PV fibers/terminals containing ChR2-EYFP (green) to TRN-PV neurons (red). Scale Bar: 10 µm. (**C**) Digitally enlarged view of the area shown in the box in B. A white arrow shows a representative case of close apposition (green/red overlap) of BF-PV fiber on a TRN-PV cell. Scale Bar: 5 µm.

Previous studies have identified prominent GABA/PV input to the TRN from the BF^26,28,29,35^. However, the functional role of this input has not been examined. To test whether we could indirectly inhibit sleep spindles through activation of this presumed inhibitory pathway, BF-PV neurons were bilaterally transduced with ChR2-EYFP, and optical cannulae were implanted targeting TRN, allowing stimulation of BF-PV fibers/terminals. Histological analysis confirmed a dense plexus of ChR2-EYFP expressing BF fibers in TRN (Fig. 5**)**. *In vivo* single-unit recordings from TRN neurons confirmed that activation of BF-PV terminals in TRN inhibited neuronal discharge (Supplemental Fig. 2), as predicted.

**Figure 6:**
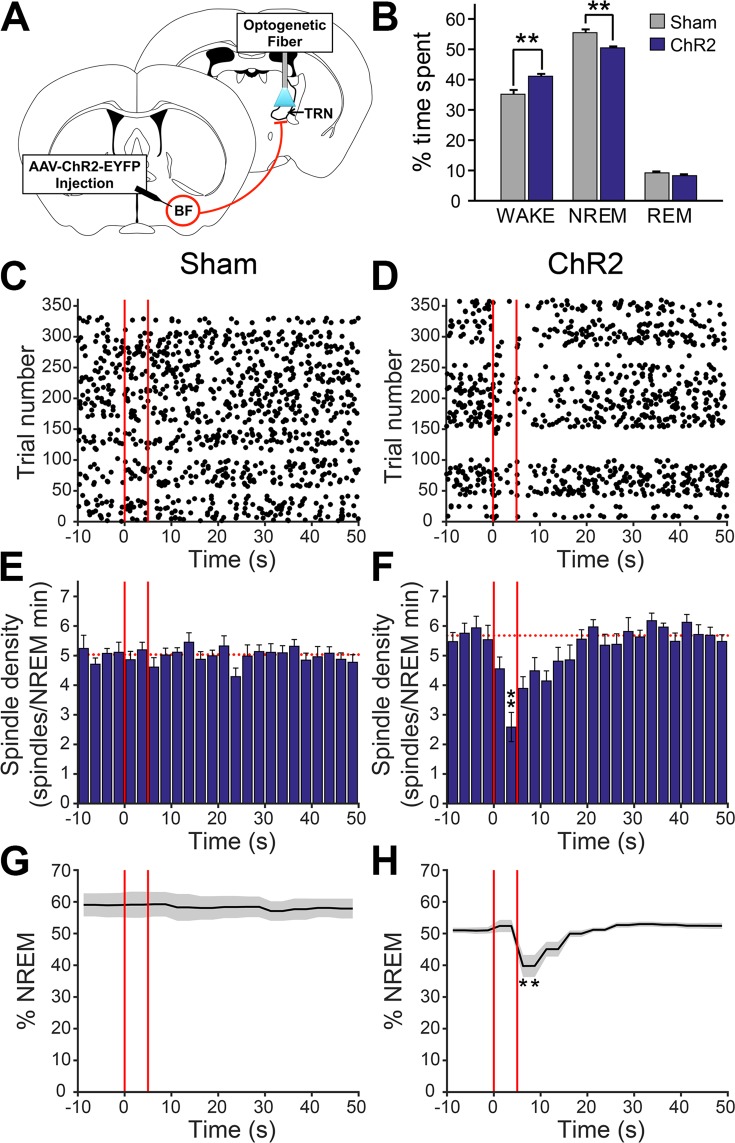
Optogenetic channelrhodopsin (ChR2) stimulation of the terminals of basal forebrain (BF) GABA/parvalbumin (PV) neurons in the thalamic reticular nucleus (TRN) reduces spindle density and decreases non-rapid eye movement (NREM) sleep. (**A**) Model of experimental design showing viral injection into BF-PV, and targeting of BF terminal extensions in TRN for optical stimulation. Coronal schematic representation of TRN and BF was adapted from The Allen Mouse Brain Atlas, (Available from: http://mouse.brain-map.org)^71^. (**B**) A statistically significant increase in cumulative wakefulness (6 h of experiment time) and decrease in NREM sleep was evident with stimulation of BF GABA/PV terminals. (**C**,**D**) Representative raster plots of detected spindle-like events relative to bilateral ChR2 stimulation or sham stimulation (red lines) show a pronounced decrease in spindle activity (**D**) compared to sham (**C**). (**E**,**F**) Histograms of averaged (n = 8) spindle density (2.5 s bins) show a decrease during stimulation of BF GABA/PV terminals (5 s/60 s) located in TRN when compared to sham stimulation. Dashed horizontal red lines additionally denote an elevation in background spindle density observed in optically stimulated mice (**F**) compared to sham stimulated (**E**). (**G, H**) ChR2 stimulations lead to an overall decrease in % of time spent in NREM sleep (**H**), compared to sham (**G**), with a pronounced drop towards the end of laser-on time and continued after the laser was turned off. *p < 0.05; **p < 0.01.

Optical excitation of BF-PV terminals in TRN at 40 Hz (10 ms pulses), a frequency within the normal discharge range of BF-PV neurons^26^ was delivered for 5 s/min for 6 h from ZT2-ZT8 (n = 8, Fig. 6A). BF-PV terminal stimulation led to an increase in total wakefulness, during 6 h stimulation (sham, 35 ± 1.4%; ChR2, 41.2 ± 0.7%, p < 0.006; Fig. 6B). The stimulation rapidly inhibited NREM spindle density, an effect which subsided within 10–15 s of stimulus cessation (Fig. 6C–F). There was a significant difference in spindle density between the sham and optical stimulation conditions and between the pre-inhibition vs. inhibition 2.5 s bins (Repeated measures 2-way ANOVA (Group, Bin) df, 23; F-ratio 3.8, p < 0.001). Interestingly, an overall increase in spindle density was also observed compared to sham stimulation controls over the entire experiment. BF-PV terminal stimulation also increased arousals from sleep within 10 s of stimulation, suggesting that a fraction of the stimulation events resulted in wakefulness (Fig. 6G,H) which also impacted the total amount of wakefulness.

To rule out that the possibility that decreased spindle density is due to change in state, we selected all BF-PV terminal stimulation trials which occurred in NREM sleep, and where the behavioral state did not change within 10 seconds from the onset of stimulation (sham, 49.0 ± 1.4% (144 ± 4.7/358 trials); ChR2, 34.6 ± 3.0% of stimulation trials (104 ± 9.9/358 trials)). Even in the absence of arousal, we still observed a robust inhibition of spindle activity (Supplemental Fig. 3A,B**)**. A concurrent decrease in power across the sigma range (10–15 Hz) was also observed in the power spectral density analysis of the 5 seconds of stimulation period compared to sham (Supplemental Fig. 3C). This analysis additionally revealed a significant increase (p < 0.05) in cortical oscillatory activity around the stimulation frequency (38.8–41.6 Hz), and a nonsignificant increase in the delta frequency range, when compared to mock-stimulation.

### Localized Inhibition of T-type Ca^2+^ Channel Activity Impairs Spindle Generation

**Figure 7:**
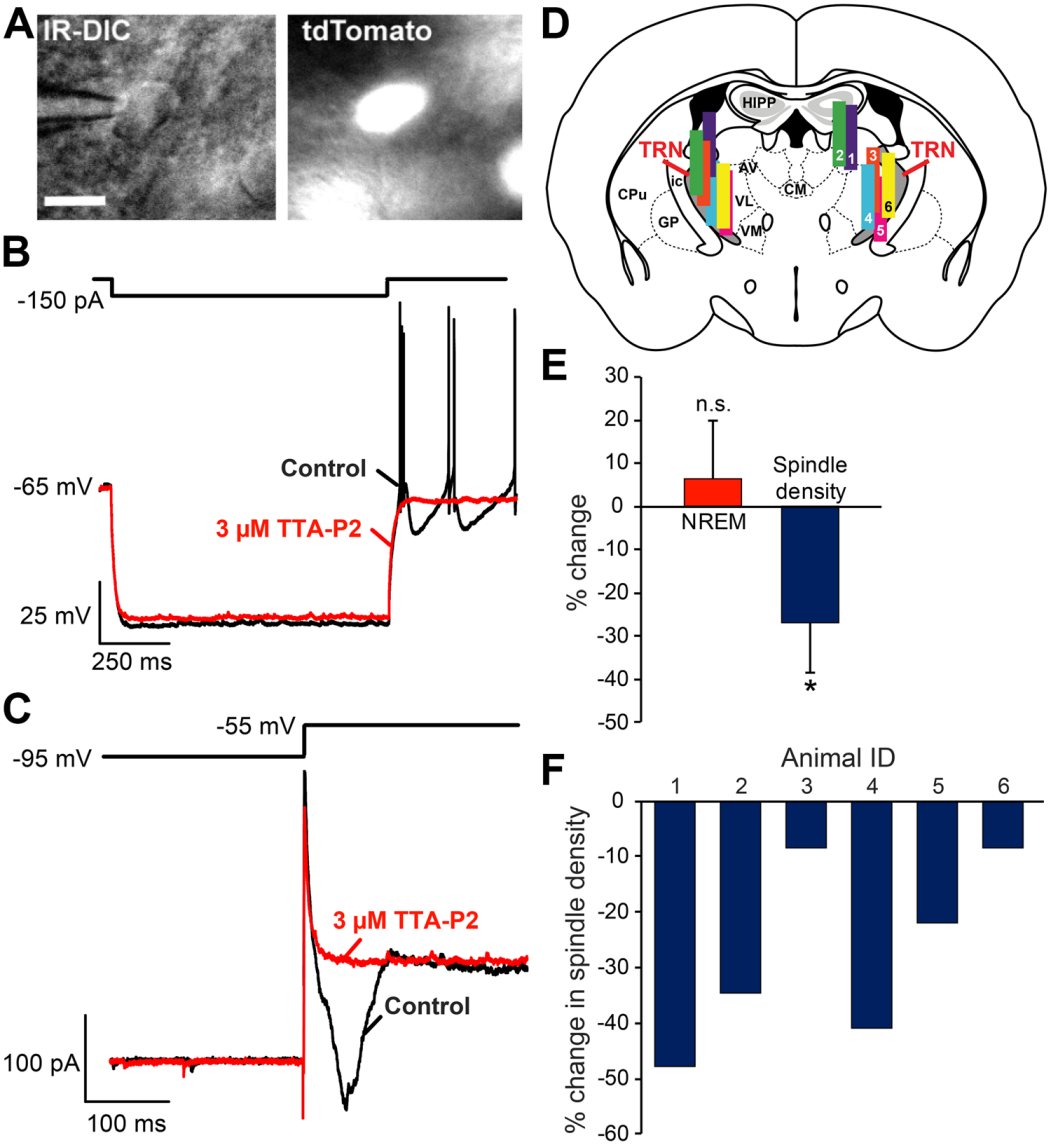
The T-type Ca^++^ channel (Ca_V_3.3) inhibitor, TTA-P2, blocks low-threshold currents in thalamic reticular nucleus (TRN) parvalbumin (PV) neurons and decreases spindle density without altering the amount of time spent in non-rapid-eye-movement (NREM) sleep. (**A**) Black and white infra-red DIC (IR-DIC) and fluorescent (td-Tomato) images of an identified PV neuron in TRN. Scale bar: 10 µm. (**B**) In current-clamp, the same neuron as shown in A exhibited low-threshold spikes following 1 s hyperpolarizing current injections (control: black trace) which were blocked by TTA-P2 (red trace). (**C**) In voltage-clamp and in the presence of 500 nM TTX, a representative TRN-PV neuron showed an inward current at the removal of a 1 s voltage step to -95 mV (control: black trace). TTA-P2 also blocked this rebound inward current (red trace). (**D**) Coronal schematic representation of TRN adapted from The Allen Mouse Brain Atlas (Available from: http://mouse.brain-map.org)^71^ provides the location of the cylindrical microdialysis membranes for each mouse in our *in vivo* studies (n = 6). (**E**) Bilateral reverse microdialysis of TTA-P2 (1 µM) into TRN from ZT2-ZT6 did not significantly affect NREM sleep but significantly decreased spindle density (n = 6, *p < 0.01). (**F**) Spindle density was reduced in every mouse receiving TTA-P2. Abbreviations: AV, anteroventral thalamic nucleus; CPu, caudate putamen; CM, centromedial thalamus; GP, globus pallidus; HIPP, Hippocampus; ic, internal capsule; TRN, thalamic reticular nucleus; VL, ventrolateral; VM, ventromedial thalamic nucleus.

Global knockout of the Ca_V_3.3 T-type calcium channel, abolished low-threshold spikes in TRN neurons and decreased spindle-frequency (sigma) activity at the transition from NREM to REM sleep^18^. However, whether the loss of spindle activity results from changes in TRN is unknown. Thus, we inhibited T-type Ca^2+^ channels in TRN, using reverse microdialysis of the selective T-type inhibitor, TTA-P2. Whole-cell patch-clamp recordings confirmed the effectiveness of this drug in blocking T-type channels in TRN-PV neurons. These neurons exhibited low-threshold spikes or inward currents after removal of hyperpolarizing currents (n = 4) or voltage steps (n = 4) respectively, which were blocked by TTA-P2 (3 µM) (Fig. 7A–C). In *in vivo* reverse microdialysis experiments, all mice tested (n = 6) with confirmed probe placement within/near TRN showed a significant decrease in spindle density compared to baseline records (-27.0 ± 11.5%; p = 0.008), with no significant change in NREM sleep (Fig. 7D–F). Delta (0.5–4 Hz) and slow-wave activity (0.75–1.5 Hz) were also unaffected.

## Discussion

In this study, we employed both optogenetic and reverse-microdialysis techniques to examine the impact of modulating TRN-PV neural activity on sleep spindles, sleep/wake behavior, and resting EEG activity. Novel findings include: (i) Optogenetic stimulation of TRN-PV neurons using a low power, waxing-and-waning stimulation paradigm elicited cortical events indistinguishable from spontaneously occurring sleep spindles with high efficiency and without altering behavioral state. (ii) Direct optical inhibition of TRN-PV neurons altered cortical oscillatory activity (delta and gamma band) associated with neuropsychiatric disorders; (iii) Spindle density was markedly reduced by optogenetic stimulation of inhibitory BF GABA/PV inputs to TRN; and (iv) Localized pharmacological inhibition of low-threshold calcium channels, implicated as a risk factor for schizophrenia by genetic studies, also significantly reduced spindle density. These findings link altered TRN activity to EEG abnormalities observed in schizophrenia and other disorders and provide optogenetic methods which will be useful in testing the role of sleep spindles in memory consolidation.

### Waxing-and-Waning Optogenetic Stimulation of TRN-PV Neurons Reliably Elicits Naturalistic Cortical Spindle Activity, Without Changes in Sleep/Wake

Several previous studies examined the effect of optogenetic stimulation of TRN neurons on cortical spindle activity and sleep/wake using different mouse models and different stimulation paradigms^30,34,36–39^. However, each of these previous studies has some methodological drawbacks. Phasic stimulation of TRN neurons at a rate close to the peak spindle frequency in mice, using relatively high (20 mW), constant, laser power increased EEG power within the sigma band^26,34^ and improved sleep-dependent memory consolidation^38^. However, the temporal profile of EEG activity produced by this pattern of stimulation does not resemble that of spontaneously occurring spindles and impacts the distribution of sleep-wake states^34^. Studies using single pulse or continuous stimulation-induced spindle-like EEG activity but with a relatively low efficiency (Halassa and colleagues reported ~19% efficiency during NREM sleep^30^, whereas we report 32.5% efficiency using single pulses). Furthermore, as reported here, many (~30%) of the laser stimulation trials elicit, brief, ‘spike-like’ EEG events, distinct from endogenously-generated sleep spindles. Similar spike-like artifacts associated with optical stimulation can also be observed in several earlier studies^36,39^.

To help avoid such artifacts and generate more naturalistic EEG spindles, we titrated the power used for optical stimulation for each animal to the lowest possible level (0.5–1.5 mW) and used a waxing-and-waning stimulation paradigm only during NREM sleep. Use of this stimulation paradigm was extremely reliable (58.6% efficiency), induced cortical responses with a temporal profile indistinguishable from spontaneously occurring spindles, and did not alter the distribution of sleep-wake states. Thus, this stimulation paradigm will be extremely useful to investigate the role of spindles in sleep-dependent memory consolidation without confounding effects on behavioral state or slow-wave activity. Furthermore, our findings support previous studies^36,40^ suggesting that increased phasic activity specifically of TRN-PV neurons is sufficient to generate spindle activity.

### Optical Inhibition of TRN-PV Neurons Does Not Block Spindles, but Impairs the 40 Hz ASSR and Increases Spontaneous Gamma and Delta Band Activity

Although many studies have now used optogenetic stimulation to induce cortical spindles, studies blocking such activity with optogenetic inhibition are scarce. Thus, we attempted to inhibit TRN-PV neurons using ArchT. Since prolonged illumination of ArchT may produce undesirable side-effects^41^, we used an intermittent illumination paradigm with the laser on for 1 min and off for 4 min. Surprisingly, this protocol resulted in neither an immediate nor an overall decrease in spindle density. In fact, spindle density tended to be enhanced progressively across the 1 min of optical inhibition. We believe that there are several potential reasons that explain this counterintuitive finding. First, it is possible that our inhibition of TRN was incomplete, and thus insufficient to completely silence neural activity and sleep spindle generation. This seems probable given that the thinness and overall architecture of the TRN make adequate light delivery and incomplete viral expression a concern^39^. Thus, these results may indicate that techniques which afford more extensive suppression of TRN are required to achieve the predicted result in this preparation (see below). It has additionally been suggested that there are two distinct subpopulations of TRN neurons, one correlated with the generation of sleep spindles and another with arousal^37^. Thus, it is possible that the targeting of our optogenetic inhibition is not focused specifically enough on the spindle generating population of TRN neurons to effectively block spindle generation.

The observed increase in spindle density during optical inhibition may stem from the complex and unique mechanisms involved in regulating cortico-thalamic network activity. For thalamic neurons, any mechanism that produces membrane hyperpolarization, whether through a reduction in excitatory drive, or increased inhibition, will trigger low-frequency rhythmicity^4^. Sufficient hyperpolarization of TRN neurons will lead to enhanced de-inactivation of T-type Ca^2+^ channels, critical for low-threshold spikes (LTS) and spindle generation. Thus, while not sufficient to completely silence TRN neuronal activity, ArchT inhibition may have resulted in moderate hyperpolarization of TRN-PV neurons, perhaps predisposing them to spindle-generating burst-firing activity in response to excitatory input. Supporting this idea, spindle oscillations have been observed to be preceded by long-lasting hyperpolarization, fostering the transition of TRN firing from tonic- to burst-firing mode^12^.

While post-hoc histology did not show any tissue damage/lesion resulting from the paradigm used for optical inhibition, we acknowledge that we cannot completely rule out the potential of side-effects/issues associated with the use of ArchT factoring in to this negative finding. First, prior reports suggest that our paradigm could increase the local temperature of brain tissue^42^. This may lead to an elevation in neuronal firing in the TRN. Second, prolonged ArchT-mediated proton pumping may lead to changes in pH and elevated intracellular Ca2+ in synaptic terminals which may increase spontaneous release of neurotransmitters as suggested by Mahn and colleagues^43^. Further, desensitization of ArchT, changes in ionic gradients, or compensatory changes in TRN during the long 1 min laser illumination may also play a role.

As suggested by Pinault and colleagues^22^, TRN-PV neurons are endowed with the state and voltage-dependent pacemaker properties to not only entrain spindle frequency activity, but also rhythmic activity in the delta and gamma band range. There is a growing body of evidence implicating impaired thalamic circuitry in a number of neurologic and psychiatric disorders, including schizophrenia^4,44^. While a number of symptoms linked to schizophrenia have been associated with dysfunction of cortical PV interneurons^20,45–47^, perhaps TRN-PV neurons deserve increased focus.

While direct optical inhibition of TRN-PV neurons did not reduce spindle activity during NREM sleep, shorter (2 s) periods of ArchT inhibition, largely during wake, did impact the animal’s ability to respond to 40 Hz steady-state auditory stimulation, reminiscent of findings in patients with schizophrenia^23,48^. Abnormal ASSR has been classically associated with a number of severe neuropsychiatric disorders, including schizophrenia and autism, which are associated with abnormal functional connectivity and highly distributed impairment in neural oscillations^3,49–51^. Further analysis revealed that this finding was due to an increase in background EEG gamma activity, likely due to widespread disinhibition of thalamic relay neurons. We also identified an elevation in spontaneous delta frequency activity with ArchT inhibition. Hyperpolarizing TRN neurons with an NMDAR antagonist *in vitro* de-inactivates T-type Ca^2+^ channels and converts the firing mode of the neuron from tonic spiking to burst-firing mode, enhancing rhythmic activity in the delta frequency range^52^, which may account for this effect. Altered background gamma activity has been linked to positive symptoms in schizophrenia^53,54^, as well as deficits in cognitive performance^51^ while altered delta is associated with negative symptoms^4^. Thus, reduced activity of TRN-PV neurons perhaps drives the TRN toward a state where pathological oscillations are prone to develop and may account for several symptom-associated electrophysiological endophenotypes of schizophrenia.

### BF-PV Terminal Stimulation in TRN Blocks Spindle Generation and Enhances Arousal

In addition to the intrinsic mechanisms described above, extra-thalamic modulatory inputs play an important role in the state-dependent modulation of thalamic network activity^5,55^. Both brainstem and forebrain arousal centers innervate the thalamic network and are believed to provide dynamic modulation of the functional interaction between TRN-thalamocortical neurons, and ascending inputs to the cortex^2,3^. Specifically, ascending extra-thalamic inputs play an important role in the state-dependent modulation of thalamocortical oscillations^56^. A previous optogenetic study found that cholinergic BF inputs influence TRN network activity^57^, but no previous study has determined the functional effect of the prominent BF GABA/PV input^26,28,29,35^. Here we showed for the first time that optogenetic stimulation of BF-PV terminals in the TRN at a frequency mimicking their discharge rate during wakefulness and REM sleep (~40 Hz)^26,58^ immediately suppressed sleep spindles, and with a longer delay, promoted transitions to wakefulness. Similar effects on arousal were found by Gutierrez-Herrera and colleagues, when optogenetically stimulating the terminals of GABAergic lateral hypothalamus in TRN^59^. Although BF-PV neurons discharge more rapidly during wakefulness and REM sleep, they do not cease firing during NREM sleep^26,58^, suggesting that they may play an active role in control of sleep spindle density, as well as promoting transitions to wakefulness and cortical gamma-band activity^26^. We believe our findings are largely consistent with this idea, as examination of stimulation trials occurring exclusively during NREM and not resulting in arousal, still showed a robust inhibition of spindle density. Thus, the observed decrease in spindle activity was not strictly due to arousal. Finally, we demonstrated that prolonged stimulation of BF-PV terminals in TRN led to an elevation in baseline spindle density. This elevation is likely linked to the frequent arousals elicited with stimulation of BF-PV terminals. Upon transition back to slow wave sleep, neuromodulatory tone gradually decreases thalamocortical neuron resting membrane potential, biasing output first to spindle generation and then to delta-activity^60^. Thus, the enhancement of sleep-to-wake transitions would be predicted to bias the thalamocortical network into a state which promotes spindle generation.

Interestingly, optogenetic stimulation of BF-PV terminals in TRN led to enhanced cortical activity at the stimulation frequency of 40 Hz. One possibility to explain this result is antidromic stimulation of cortically projecting BF neurons, which as we previously reported can modulate cortical gamma^26^. Alternatively, synchronous rhythmic inhibition of TRN neurons may entrain the firing of thalamocortical neurons.

### Local Inhibition of TRN T-type Calcium Channels Inhibits Spindles Without Altering NREM Sleep Time

The *CACNA1i* gene encoding Ca_V_3.3 T-type calcium channels was implicated as a prominent risk gene by large scale genetic studies of Schizophrenia^17^. Furthermore, a *de novo* mutation of this gene observed in a patient with schizophrenia led to reduced low-threshold calcium currents in an expression system^16^. Constitutive knockout of Ca_V_3.3 channel expression attenuates spindle-frequency EEG activity at the transition from NREM to REM sleep and leads to fragmented sleep^18,61^. However, these findings do not directly implicate the TRN in these deficits since Ca_V_3.3 channels are expressed in cortical interneurons^62^ and elsewhere in the brain. Thus, here, we used reverse microdialysis to locally inhibit T-type calcium channels in TRN, and report that the selective T-type calcium channel antagonist, TTA-P2, significantly reduced cortical spindle density in frontal cortex EEG recordings, while having no significant effect on sleep/wake behavior or slow-wave activity. These results differ slightly from a recent publication by Fernandez *et al*.^63^, who reported no change in spindle density, but described highly localized changes in spindle amplitude and intra-spindle frequency only in somatosensory cortex (S1 and S2), without any change in the prelimbic cortex of the Ca_V_3.3 constitutive knock out mice. Although it is possible that minor differences in electrode location and impedance or spindle detection parameters could explain these differences, the global absence of the channel in the entire brain, including the cortex in the Fernandez *et al*., study, in the constitutive Cav3.3 knockout mice as well as the possibility of developmental compensation could also affect the results. We note that like Fernandez and colleagues, our EEG recordings predominantly detected local spindles (Supplemental Fig. 4).

Several lines of evidence suggest our observations are due to a localized blockade of T-type channels in the TRN: 1) Spindles were only affected when microdialysis probes were located within or close to the TRN bilaterally; 2) Slow-wave activity was unaffected by perfusion of TTA-P2, in contrast to previous findings when TTA-P2 was infused into the relay thalamus^64^; and (3) Knockout of the Ca_V_3.1. channel present in relay neurons, which is also blocked by TTA-P2, did not alter spindle activity^65^. Thus, our findings provide supportive evidence for a localized reduction in Ca_V_3.3. activity in TRN as a cause of spindle abnormalities in schizophrenia.

## Conclusions

Together with recent clinical findings^8,66^, our optogenetic and pharmacological data support downregulation of TRN-PV neuronal activity as a contributor to the EEG abnormalities typical of schizophrenia, especially sleep spindle deficits. Further, these data provide important mechanistic insights for understanding the role of TRN-PV neurons regulating thalamic network activity. Our findings show that use of optical stimulation where power is modulated with a waxing-and-waning spindle-like profile efficiently evoked NREM spindle-like events which were morphologically similar to physiological spindles, without altering behavioral state. Moving forward, this paradigm, in combination with more localized recording techniques, will provide a powerful tool to investigate the role of sleep spindles, dissociated from effects on sleep, in cognitive function.

In this study, we focus exclusively on the TRN-PV neurons, as they represent the predominant population of neurons in TRN, exhibit large T-type calcium currents and are more prone to generate the rhythmic bursting which underlies sleep spindle generation^40^. However, we note that another major subpopulation of GABAergic neurons, which express SOM, is present in this brain region, and is likely to also play a significant role in the complex functionality of the thalamic network. Recent work showed that deletion of a schizophrenia-related gene, ErbB4 from TRN-SOM neurons impairs sensory selection^19^.

Indirect modulation of TRN activity via stimulation of BF-PV terminals in TRN strongly impacted sleep and spindle activity. These findings show that, in addition to direct cortical projections^26^, BF-PV neurons promote arousal and suppress NREM sleep^58^ through projections to TRN-PV neurons. Finally, in severe neuropsychiatric disorders such as schizophrenia, cortical PV neuron dysfunction is central to the development of a number of symptom classes^45^. Recent work suggests that this impairment may not be specific to the cortex and could involve GABA/PV neurons in subcortical regions^46^, including the TRN^14^. Our findings support this idea and implicate the TRN and BF-PV neurons as potential targets for novel therapeutic intervention for neuropsychiatric disorders.

## Methods and Materials

### Animals

Adult (4–8 months) PV-cre mice (Jackson Labs, Bar Harbor, ME; Strain# 008069) were housed with lights-on 7:00AM-7:00PM. For *in vitro* experiments PV-cre mice were crossed with Cre-reporter mice (Jackson Labs, Strain# 007905) to express the red fluorescent protein tdTomato in PV neurons. All procedures were in accordance with VA & National Institutes of Health guidelines and were approved by the VA Boston Healthcare System Institutional Animal Care and Use Committee.

### Viral Vectors

Adeno-associated viral vectors (serotype 5) were obtained from University of North Carolina Vector Core, Chapel Hill, NC. AAV-DIO-ChR2-EYFP, for Cre-dependent expression of ChR2 and the enhanced yellow fluorescent protein (EYFP), was used for excitation of TRN-PV neurons and BF-PV terminals in TRN. AAV-EF1a-DIO-ArchT-GFP, with Cre-dependent expression of ArchT and the green fluorescent protein (GFP), was used for inhibition of TRN-PV neurons. AAV-GFP control injections were also performed in two PV-tdTomato animals for confirmation of viral transduction (Fig. 1). The efficiency of transduction was similar in TRN (83 ± 3%) and BF (85.5 ± 5.5%) and is compatible with other published studies (94%)^67^.

### Stereotaxic Surgery

Surgeries were conducted under isoflurane anesthesia. For optogenetics, 500–1000 nl of viral vector was stereotaxically injected into rostrodorsal TRN (AP -0.7 mm, ML 1.3–1.5 mm, DV 3.2–3.5 mm) or BF (AP 0.0; ML 1.6 mm; DV 5.2 mm) using a glass pipette and Nanoliter 2010 injector (WPI), or a 5 µl Hamilton Syringe and Legato 130 syringe pump (KD scientific, Holliston, MA). EEG electrodes were implanted bilaterally above the frontal cortices (AP 1.5–1.9 mm; ML ± 1.0–1.5 mm), a reference screw above the cerebellum, and EMG electrodes inserted in the nuchal muscle. Fiber-optic-cannulae (MFC_200/245–0.37_10mm_ZF1.25(G)_FLT, Doric Lenses, Quebec, Canada) were bilaterally implanted targeting TRN. For reverse microdialysis experiments, CMA 7 guide cannula (Harvard Biosciences Inc., Holliston, MA) were implanted bilaterally targeting TRN. Mice were allowed at least one week to recover before experiments began.

### *In Vivo* Electrophysiological Recordings and Data Analysis

Recordings were performed in freely moving animals, following 2 days of habituation. EEG/EMG signals were filtered (0.5–200 Hz) and sampled at 2–4 kHz using Spike2 (Cambridge Electronic Design, Cambridge, UK), with an A&M systems amplifier and CED 1401 digitizer or SireniaPro with Pinnacle amplifier (Pinnacle Technologies, Inc., Lawrence, KS), and WinWCP (University of Strathclyde, Glasgow, UK) software. Optogenetic stimulation was performed using a 473 nm laser (DL473–80–0, Crystalaser Inc., Reno, NV) for ChR2, or 532 nm laser (MGL-III-532–100mW; Opto Engine, Midvale, UT) for ArchT. Laser light was delivered to TRN via a fiberoptic patch cable (MFP_200/220/900–0.22_2m_FCM-MF1.25; Doric Lenses). All optogenetic manipulations were compared with ‘sham’ controls (TTL pulses with laser off). Auditory stimulation was delivered via a cage mounted speaker (~85 dB). In order to restrict stimulation to NREM sleep, a NREM detection system (NREM gate) was developed by utilizing open source software (Bonsai, OpenEphys.org), for real-time monitoring of EEG delta power via an Arduino Uno board (Arduino LLC, www.arduino.cc), and permitted threshold based discrimination of state. Sleep scoring was performed using Spike2 or SireniaPro software, with further analysis done using MATLAB (R2016a, MathWorks, Natick, MA) or Igor Pro (Wave Metrics, Lake Oswego, OR). Spectral analysis of data was performed using the multitaper method (Chronux Toolbox; http://chronux.org/), to reduce signal bias and variance^68^.

Sleep spindles were detected using an automated algorithm developed in-house (MATLAB)^31^ (Fig. 1C–F). Briefly, EEG data was band-pass filtered (10–15 Hz, Butterworth Filter) and the root-mean-squared (RMS) power calculated to provide an upper envelope of the data. The RMS data was then exponentially transformed to further accentuate spindle-generated signals over baseline. Putative spindle peaks were identified in transformed data via crossing of an upper-threshold value, set as 3.5x the mean RMS EEG power across all states for each mouse. Additional detection criteria included a minimum duration of 0.5 s, based on crossing of a lower threshold set at 1.2x mean RMS power, and a minimum inter-event interval of 0.5 s. This automated spindle detection algorithm has been rigorously tested in comparison to manual spindle detection. In order to determine the ratio of spindles that co-occurred in more than one cortical area, in 3 mice we assessed the number of spindles that were simultaneously detected in frontal and parietal EEG recordings and observed only 25% of spindle-co-occurrence (see Supplemental Fig. 4) which is consistent with the previous report^69^, suggesting that our frontal EEG electrode is capable of capturing both localized as well as global spindle activity.

### *In vivo* Reverse Microdialysis

Reverse microdialysis of 5-dichloro-N-[1-(2,2-dimethyl-tetrahydro-pyran-4-ylmethyl)-4-fluoro-piperidin-4-ylmethyl]-benzamide (TTA-P2) bilaterally into TRN was performed for 4 h during the light period (ZT2-ZT6). Dose-finding studies (1, 10, 30, 100 & 300 µM) determined the optimal dose to be 1 µM. Microdialysis probes were inserted into cannulae 16 h prior to the experiment. On Day 1, artificial cerebrospinal fluid (aCSF) was infused (control). On Day 2, TTA-P2 (1 µM) was infused and the effect on NREM spindle density was compared with aCSF control. David and colleagues estimated the spread of the drug at a concentration of 1 µM to be ~200 µm from the point of delivery^64^, which here included the targeted rostrodorsal area of TRN.

### *In vitro* Whole-Cell Patch Clamp Recordings

Coronal brain slices containing TRN (Bregma -0.46 to -0.94 mm) were prepared using standard techniques^26,70^. Slices were perfused with warmed aCSF, in mM 124 NaCl, 1.8 KCl, 25.6 NaHCO_3_, 1.2 KH_2_PO_4_, 2 CaCl_2_, 1.3 MgSO_4_ and 10 glucose, osmolarity 300 mOsm, saturated with 95% O_2_/5% CO_2_ at 32 °C). Whole-cell recordings of fluorescent (tdTomato and/or GFP conjugated to AAV-ArchT) TRN-PV neurons were made using glass patch pipettes (3–6 MΩ) filled with intracellular solution containing (in mM): 130 potassium gluconate, 5 NaCl, 2 MgCl_2_, 10 HEPES, 0.1 EGTA, 2 Na_2_ATP, 0.5 NaGTP, 4 MgATP, 1 spermine, 0.5% biocytin (pH 7.25 with KOH, 280 mOsm). Signals were recorded using a Multiclamp 700A amplifier with pClamp 10.0 software (Axon Instruments, San Jose, CA), at a sampling rate of 50 kHz and low-pass filtered at 10 kHz. Drugs were bath-applied. For optogenetic inhibition, 532 nm light was delivered using a X-Cite 120 fluorescence illumination system (Excelitas Technologies Corp, Waltham, MA) through a 40× water-immersion objective (~5 mW).

### Immunohistochemistry

Mice were perfused transcardially with 10% formalin. Brains were post-fixed for 1–2 days and transferred to 30% sucrose for 2–3 days. Coronal slices were cut (40 µm-thickness) and stored in phosphate-buffered saline at 4 °C. For anti-GFP & PV labeling, sections were incubated in mouse anti-GFP (1:1000; Cat.#MAB3580; MilliporeSigma, Burlington, MA) followed by incubation with secondary donkey anti-mouse-AlexaFluor488 (green; 1:100; Cat#A21202; ThermoFisher Scientific, Waltham, MA). Subsequently, slices were incubated in sheep anti-PV (1:200; Cat. #AF5058; RnD Systems, Minneapolis, MN) and then secondary donkey anti-sheep-AlexaFluor594 (red; 1:100; Cat.#A11016; ThermoFisher Scientific). Fluorescence microscopy was performed using either a Nikon eclipse inverted confocal microscope and NIS-element imaging software or Zeiss Image2 microscope with Neurolucida software (Microbrightfield; Williston, VT). Fiber-optic cannula and dialysis probe locations, determined using cresyl violet staining, were mapped onto appropriate schematic templates from The Allen Mouse Brain Atlas Allen Institute for Brain Science. ©2011. Available from: http://mouse.brain-map.org ^71^.

### Statistics

Data are presented as mean ± standard error. Statistical analysis of spindle and sleep/wake data was performed using JMP pro12 (SAS Institute Inc., Cary, NC). Paired comparisons of means were evaluated using Student’s t-test. Multiple mean comparisons were performed using repeated measures ANOVA and Turkey HSD post-hoc analysis. The power spectra were evaluated using the Jackknifing U-statistical test to mitigate effects of artifacts and intermittent outliers in data^72^.

## Supplementary information

Supplementary Information

## Data Availability

All data generated or analyzed during this study are included in this published article (and its Supplementary Information files).
