# Supplementary material for: Thalamic Reticular Nucleus Parvalbumin Neurons Regulate Sleep Spindles and Electrophysiological Aspects of Schizophrenia in Mice
Source: Sci Rep. 2019 Mar 5;9:3607. doi: 10.1038/s41598-019-40398-9 (PMC6401113; doi:10.1038/s41598-019-40398-9)
Supplement: Supplementary file 1 — Supplementary Information [file 41598_2019_40398_MOESM1_ESM.pdf]

**Supplementary Information**

# Thalamic Reticular Nucleus Parvalbumin Neurons Regulate Sleep Spindles and Electrophysiological Aspects of Schizophrenia in Mice

***Authors:***

Stephen Thankachan[1#], Fumi Katsuki[1#], James T. McKenna[1], Chun Yang[1], Charu Shukla[1], Karl Deisseroth[2], David S Uygun[1], Robert E. Strecker[1], Ritchie E. Brown[1], James M. McNally[1*], Radhika Basheer[1*]

**Supplementary Figure S1**

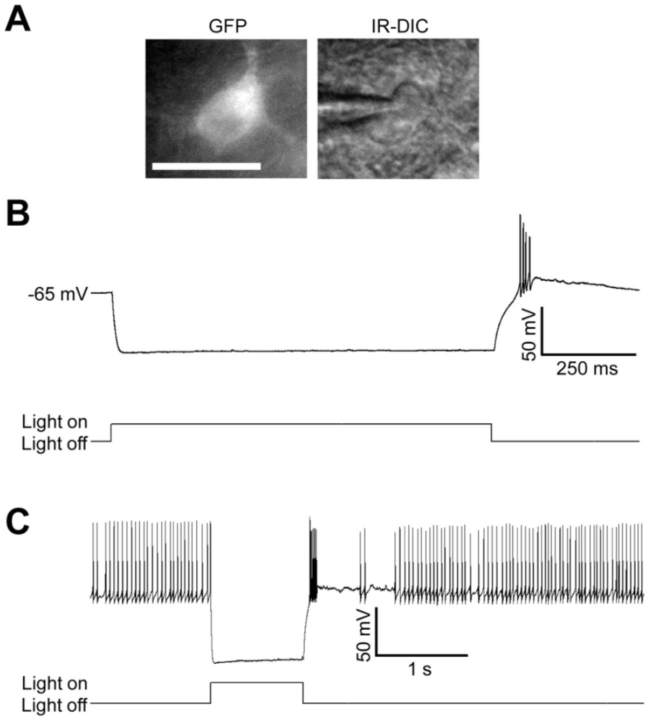


**Supplementary Figure S1. *In vitro* recordings confirmed activation of ArchT hyperpolarized TRN-PV neurons, and inhibited action potential discharge. (A)** Black and white Fluorescent (GFP) and infrared differential interference contrast (IR-DIC) images of a representative TRN AAV-ArchT-GFP expressing PV neuron. Scale bar: 25 µm. **(B)** Whole-cell recording from the same neuron in A showed that a 1-s green light pulse (530 nm) (bottom trace) strongly hyperpolarized the recorded neuron (top trace. -54.0 ± 6.7 mV, n=4), and induced a rebound action potential at the offset of the light pulse (n=4) likely due to activation of T-type Ca channels. **(C)** The same neuron as in A was induced with tonic firing by a current injection. A 1-s green light pulse prevented the ongoing action potential discharge (21 Hz). A rebound burst (90 Hz) was observed within 0.2 s after the light offset, followed by a delayed return to the baseline, likely because of delayed deactivation of ArchT. n=1.

**Supplementary Figure S2**

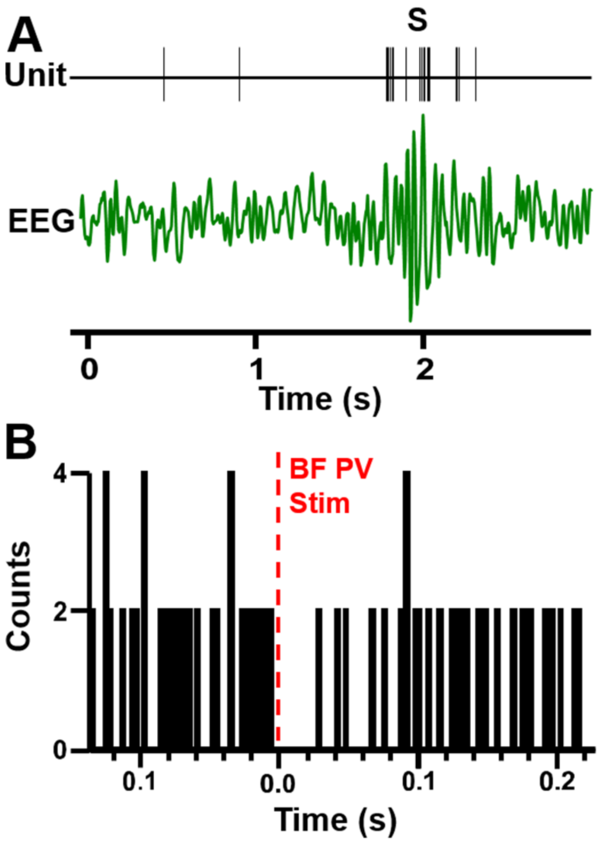


**Supplementary Figure S2. Basal forebrain (BF) parvalbumin (PV) neurons inhibit the thalamic reticular nucleus (TRN) PV neurons: Single unit recording. (A)** TRN unit recording demonstrates increased firing associated with a cortical spindle. **(B)** PV neuronal stimulation in BF leads to inhibition of the TRN unit firing (20 trials), consistent with the predicted role of the BF-PV neurons in modulating TRN activity.

**Supplementary Figure S3**

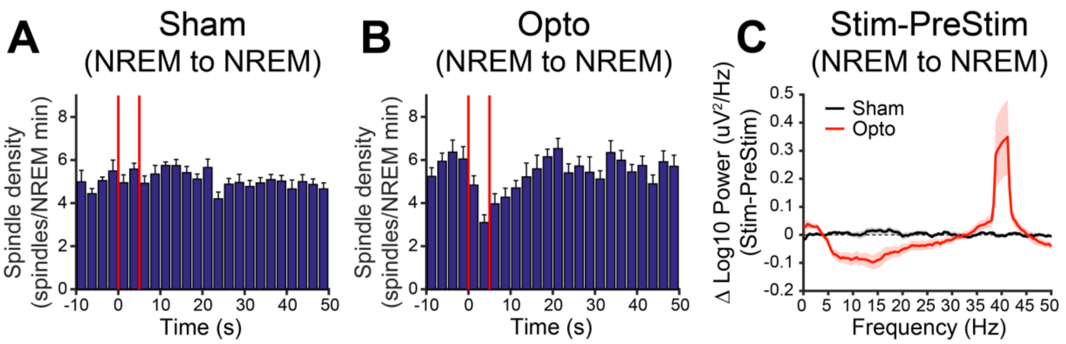


**Supplementary Figure S3. Sigma power and spindle density were reduced with the terminal stimulation of basal forebrain (BF) parvalbumin (PV) neurons in the thalamic reticular nucleus (TRN).** We specifically evaluated BF-PV terminal stimulation trials which occurred when the mouse was in NREM sleep at least for 10s prior to the stimulation (NREM gate), and where the behavioral state did not change within 10 s from the onset of stimulation was analyzed. **(A-B)** Spindle density was plotted for sham (A) and opto (B) conditions. A robust inhibition of spindle activity was observed with BF-PV terminal stimulation even without arousal. **(C)** Power spectral density (PSD) analysis of the EEG activity during BF-PV terminal stimulation in NREM trials without state change was performed by subtracting PSD of pre-stimulation period (5 s) from PSD of stimulation period (5 s). If there is no change in power between pre-stimulation and stimulation periods, the values will be zero (horizontal dotted line). Power across the sigma range decreased with stimulation (opto) compared to sham. There were large increases in cortical oscillatory activity specifically at the stimulation frequency of 40 Hz, and limited changes in the delta frequency range

**Supplementary Figure S4**

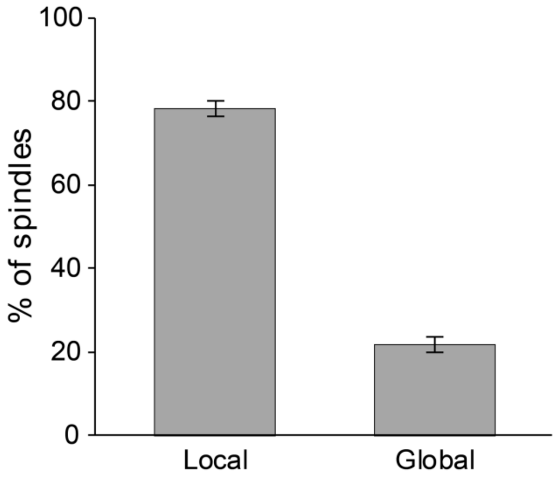


**Supplemental Figure 4. EEG electrodes capture both local and global spindles.** To assess whether our frontal EEG recordings detected region-specific (local) or globally distributed (global) spindles or both, we analyzed 6h of EEG data recorded from mice that had EEG electrodes implanted over the frontal cortex and the parietal cortex ipsilaterally (n=3). If a spindle detected in one electrode had at least 90% duration overlap with a spindle simultaneously detected in the other electrode, it was considered as a co-occurring (global) spindle. Then the percent of co-occurring spindles in relation to all detected spindles in each electrode was computed. We found that 22% of spindle events recorded in frontal cortex overlapped with those recorded in parietal cortex (i.e were global) whereas 78% of the spindles detected in the frontal EEG electrode were region specific (local spindles).
